# Nanogenerators consisting of direct-grown piezoelectrics on multi-walled carbon nanotubes using flexoelectric effects

**DOI:** 10.1038/srep29562

**Published:** 2016-07-13

**Authors:** Jin Kyu Han, Do Hyun Jeon, Sam Yeon Cho, Sin Wook Kang, Sun A. Yang, Sang Don Bu, Sung Myung, Jongsun Lim, Moonkang Choi, Minbaek Lee, Min Ku Lee

**Affiliations:** 1 Department of Physics, Chonbuk National University, Jeonju 54896, Korea; 2 Thin Film Materials Research Center, Korea Research Institute of Chemical Technology (KRICT), Daejeon 34114, Korea; 3 Research Institute of Physics and Chemistry, Chonbuk National University, Jeonju 54896, Korea; 4 Department of Physics, Inha University, Incheon 22212, Korea; 5 Nuclear Materials Development Division, Korea Atomic Energy Research Institute, Daejeon 34057, Korea

## Abstract

We report the first attempt to prepare a flexoelectric nanogenerator consisting of direct-grown piezoelectrics on multi-walled carbon nanotubes (mwCNT). Direct-grown piezoelectrics on mwCNTs are formed by a stirring and heating method using a Pb(Zr_0.52_Ti_0.48_)O_3_ (PZT)-mwCNT precursor solution. We studied the unit cell mismatch and strain distribution of epitaxial PZT nanoparticles, and found that lattice strain is relaxed along the growth direction. A PZT-mwCNT nanogenerator was found to produce a peak output voltage of 8.6 V and an output current of 47 nA when a force of 20 N is applied. Direct-grown piezoelectric nanogenerators generate a higher voltage and current than simple mixtures of PZT and CNTs resulting from the stronger connection between PZT crystals and mwCNTs and an enhanced flexoelectric effect caused by the strain gradient. These experiments represent a significant step toward the application of nanogenerators using piezoelectric nanocomposite materials.

Nanogenerators (NGs) for renewable energy harvesting are receiving increasing attention, with researchers exploring a number of possible natural energy sources including thermoelectricity, solar energy, and mechanical movement. Among these, mechanical movement is advantageous because it can provide energy continuously without any environmental restrictions. Several NGs have been developed for mechanical energy harvesting through the use of nanoscale piezoelectric materials such as ZnO NW^1^, Pb(Zr,Ti)O_3_ (PZT) nanowires^2^, Pb(Mg,Nb)O_3_-PbTiO_3_ (PMN-PT) nanowires^3^, and BaTiO_3_ (BTO) nanotubes^4^. Recently, composite-type NGs consisting of nanoscale piezoelectric materials and flexible one-dimensional nanostructures have been fabricated to enhance endurance and flexibility^5^,^6^,^7^,^8^. However, the output power of composite-type NGs needs further improvement for practical applications.

Enhanced electromechanical coupling is predicted at the nanoscale due to the flexoelectric effect, caused by inhomogenous strain or a strain gradient^9^. Although the flexoelectric coefficient is very small in bulk materials (~10^−10^ C m^−1^), it becomes more significant below 100 nm (~10^−5^ C m^−1^). Enhancement of piezoelectricity by the flexoelectric effect was observed experimentally in buckled PZT nanoribbons (a few hundred nm thick)^10^. Control of the internal electric field in BiFeO_3_ thin films due to the flexoelectric effect has been achieved^11^. However, not much work has been done to investigate the origin of the flexoelectric effect in nanomaterials, or to exploit it in real-life applications such as renewable energy harvesting.

In this letter, a simple, cost-effective synthesis method for flexible composite-type NGs is demonstrated utilizing PZT directly-grown on mwCNTs (PZT-CNTs). Mechanical deformation in our PZT-CNTs by a Teflon-disk-equipped tapping machine generated a maximum output voltage and current of 8.6 V and 47 nA, respectively. The output voltage and current is about ten times higher than NGs using simply mixed composites with crystallized PZT and mwCNTs. In order to understand the enhancement of the output power, we investigated the atomic-scale unit cell orientation and strain distribution of epitaxial PZT nanoparticles grown directly on mwCNTs (PZT NP-CNTs) prepared by filtering the PZT precursor and mwCNT mixed solution. PZT-CNT NGs, together with the soft nature of our CNT devices, will pave the way for a future application such as mobile and wearable electronics.

## Results

### Synthesis and characterization of PZT NP-CNTs and PZTs-CNTs

**Figure 1:**
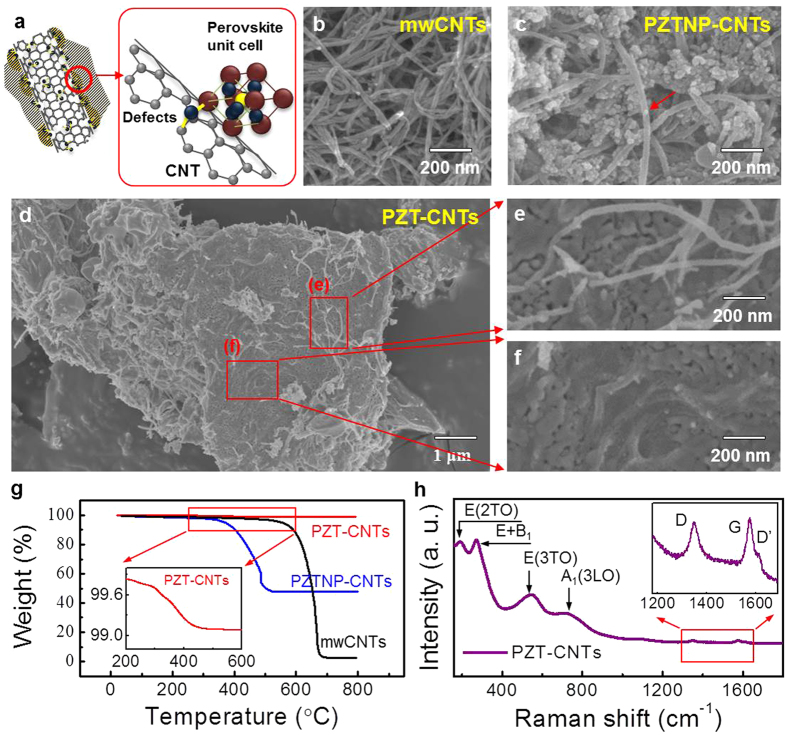
Synthesis and characterization of PZT NP-CNTs and PZT-CNTs. (**a**) Schematic diagram of PZT-CNTs. FESEM images of (**b**) mwCNTs, (**c**) PZT NP-CNTs, and (**d**–**f**) PZT-CNTs. (**g**) Thermogravimetry curves of mwCNTs, PZT NP-CNTs, and PZT-CNTs during heat treatment. (**h**) Raman spectrum of PZT-CNTs indicating the perovskite phase of PZT.

Figure 1a shows a schematic diagram illustrating PZT-CNTs composed of mwCNTs with defects including carboxylic groups (-COOH), PZT NPs, and PZT crystals. Details of the synthesis process are shown in Supplementary Fig. S1. PZT NPs nucleate at defect sites on the mwCNTs, and PZT crystals grow on the PZT NPs. Defects on mwCNTs are formed by an acid treatment which alters the mwCNT surface to have a hydrophilic nature (Supplementary Fig. S2)^12^. Here, the carboxylic group plays a key role by inducing the crystallization of PZT onto the mwCNT surface during the PZT coating process^13^. Morphological characterizations of mwCNTs, PZT NP-CNTs, and PZT-CNTs are shown in Fig. 1b–d, respectively. PZT NPs decorate the surface of mwCNTs as shown in Fig. 1c. Figure 1d shows PZT-CNTs consisting of approximately 5-μm PZT crystals and PZT NP-CNTs incorporated into the PZT crystals. PZT NP-CNTs are observed on the outside of the crystals (Fig. 1e) as well as inside the crystals (Fig. 1f), which indicates that the PZT NP-CNTs are uniformly dispersed. The loading of PZT on mwCNTs is obtained from thermogravimetric analysis (TGA) as shown in Fig. 1g. Above 600 °C, the oxidation temperature of mwCNTs, the PZT content of PZT NP-CNTs and PZT-CNTs was 44.94 ± 1.78% and 99.03 ± 0.03%, respectively, which is highlighted by the near-oxidation of mwCNTs. Figure 1h presents Raman spectra of PZT-CNTs, which provides a sensitive way to detect tetragonal symmetry in crystals with the perovskite structure. Tetragonal PZT belongs to the space group *P4 mm*, so 3A_1_ + B_1_ + 4E modes are found in its Raman spectrum. Raman peaks at 205, 275, 533, and 724 cm^−1^ are observed, corresponding to E(2TO), E + B_1_, E(3TO), and A_1_(3LO) of PZT, respectively. These phonon modes are typical Raman peaks of the perovskite PZT phase^14^. Therefore, we can confirm the tetragonality of the PZT crystals in PZT-CNTs. Moreover, bands at 1330, 1581, and 1616 cm^−1^ are observed, corresponding to disordered *sp*^2^ carbon in the mwCNTs (denoted by the D band), well-ordered graphite structures (denoted by the G band), and end planes of graphene layers (denoted by the D′ band), respectively^15^.

**Figure 2:**
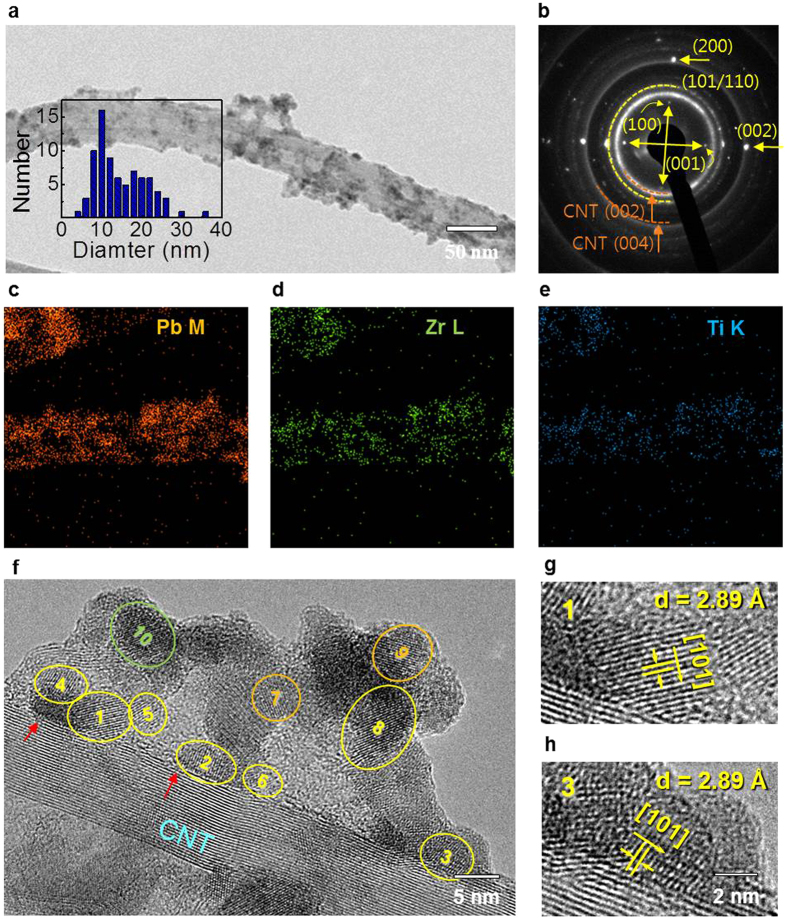
Morhpology and structure of PZT NP-CNTs. (**a**) FETEM images and (**b**) SAED patterns of PZT NP-CNTs. The inset histogram shows the diameter distribution of PZT NPs. (**c**–**e**) EDS mapping on Pb, Zr, and Ti, respectively. (**f**) HRTEM images of the PZT NP-CNTs and (**g**,**h**) magified images showing the crystal growth direction with (101) perovskite of the PZT NPs as marked 1, 3, respectively.

Field-emission transmission electron microscopy (FETEM) was performed for elemental structural analysis of PZT NP-CNTs (Fig. 2a). The diameter distribution of the PZT NPs is shown in the left inset of Fig. 2a, and the average diameter is 14.0 nm. Crystallinity was investigated by selective area electron diffraction (SAED) as shown in Fig. 2b. In the SAED patterns, reflections at 3.42 Å and 2.11 Å correspond to the lattice spacings of the mwCNT (002) and (004) planes. The measured lattice spacings of 4.14, 4.03, 2.89, 2.85, 2.07, and 2.01 Å in the NPs correspond to the PZT perovskite (001), (100), (101), (110), (002), and (200) crystal planes, respectively (JCPDS No. 33-0784), indicating that the NPs are polycrystalline. Energy dispersive X-ray spectroscopy (EDS) mapping of Pb, Zr, and Ti was performed on the PZT NP-CNTs (Fig. 2c–e) using scanning transmission electron microscopy. Pb, Zr, and Ti are clearly identifiable on the NPs confirming that they are PZT. High-resolution TEM (HRTEM) images were taken to understand the detailed crystallographic growth behavior of PZT NP-CNTs. In Fig. 2f, HRTEM reveals a well-defined PZT NP geometry on mwCNTs and clear lattice reflections. The polycrystalline nature of the PZT NPs was clearly observed, as also indicated by SAED patterns. Here, defects can be found on the surface of mwCNTs (indicated by red arrows), and we see that PZT NPs nucleate at the defects. The PZT NPs have lattice reflections corresponding to the (101) and (110) planes of the perovskite PZT phase and the (331) plane of the pyrochlore PZT phase (Supplementary Table S1). The nearest PZT NPs to the CNT surface, especially, are mainly comprised of (101) planes of the perovskite PZT phase as shown in Fig. 2g,h. Despite the high temperature annealing in nitrogen atmosphere, the ratio of oxygen-deficient secondary phase in the PZT NPs is very low. We believe that one of the reasons is the diffusion of carbonyl group, formed on the surface of mwCNTs during the functionalization process of mwCNTs, into PZT NPs (Supplementary Figs S3 and S4)^16^.

### Fabrication and characterization of NGs using PZT-CNTs

**Figure 3:**
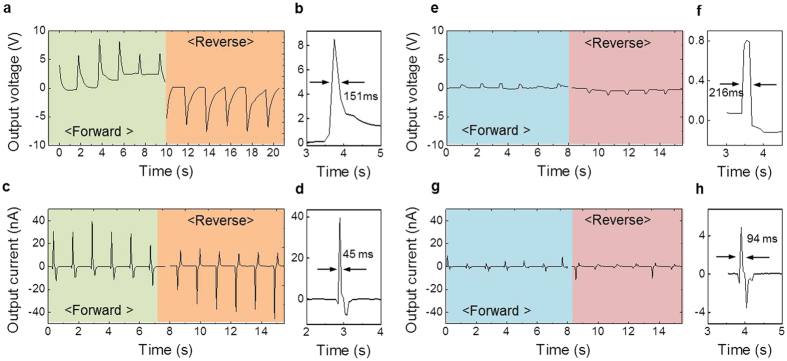
NG property of PZT-CNTs and blended-PZT-CNTs. (**a**,**c**) Output voltage and (**e**,**g**) current generated from PZT-CNTs and blended-PZT-CNTs, respectively. Magnified (**b**,**d**) output voltage and (**f**,**h**) current from PZT-CNTs and blended-PZT-CNTs.

In order to verify the NG properties, flexible films containing PZT-CNTs were sandwiched between two Au/Cr-coated polyimide layers (Supplementary Fig. S5). We measured the open-circuit voltage and short-circuit current of PZT-CNT films during periodic tapping as shown in Fig. 3. The output signals were generated by a compressive force of 20 N. Figure 3a,c show that the voltage and current reach ~8.6 V and ~47 nA, respectively. Voltage and current were also measured in reverse to verify that the signal was generated by the piezoelectric property of the PZT-CNT films, and when the connection was switched the polarity of the output signal was inverted. The output voltage and current exhibit lifetimes (determined by the full width at half maximum) of 151 ms and 45 ms, which are shown in Fig. 3b,d, respectively. Compared to blended films with PZT powder and mwCNTs (blended-PZT-CNTs), the output voltage and current of PZT-CNT films was enhanced by a factor of 10, and the lifetimes of their output voltage and current were reduced by 30% and 52%, respectively. This enhancement is most likely caused by the improved contact between PZT crystals and mwCNTs. Therefore, we conclude that the direct growth of PZT crystals onto mwCNTs enhances the output voltage and current, and reduces the lifetime of signals of NGs based on PZT-CNT relative to blended-PZT-CNT NGs.

### Atomic-scale unit cell distribution of PZT NP-CNTs

**Figure 4:**
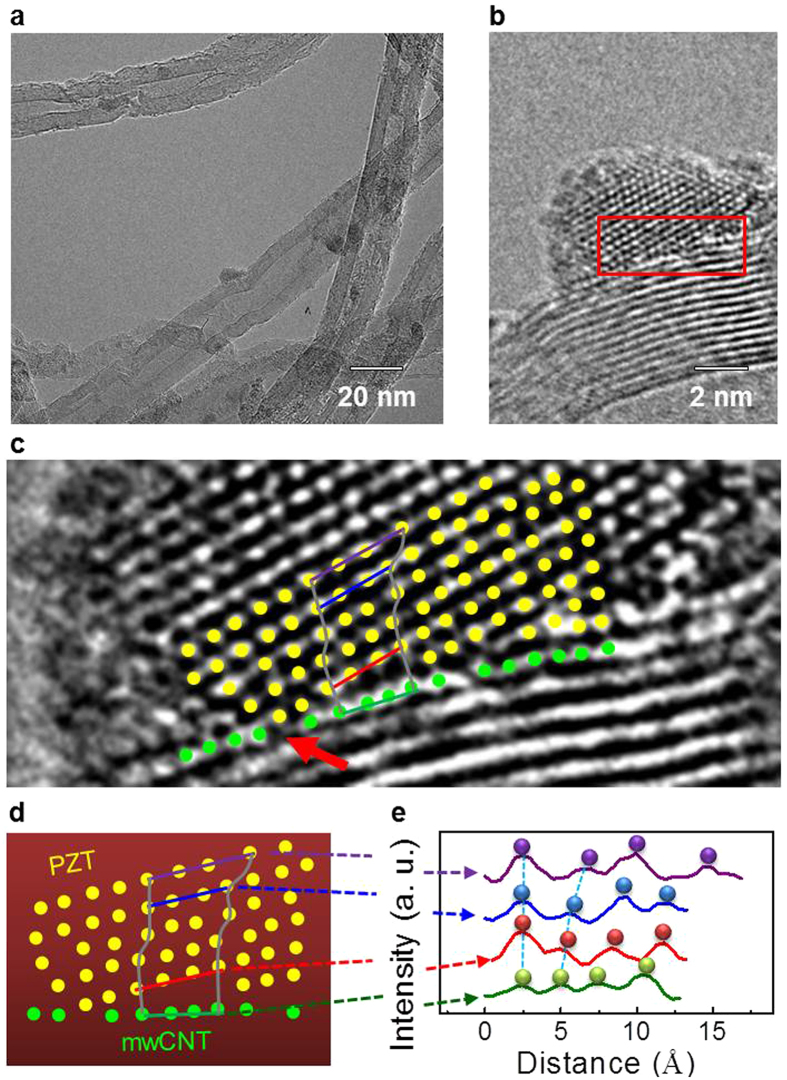
Atomic-scale unit cell distribution of PZT NP-CNTs. (**a**) HRTEM images of epitaxial PZT NP-CNTs, (**b**) magnified image of the PZT NPs, and (**c**) magnified image showing atomic arrangements. (**d**) Atomic arrangement and (**e**) lattice spacings of PZT and mwCNTs.

In order to find the origin of the enhanced NG properties, we prepared epitaxial PZT NPs on mwCNTs by controlling the molarity of PZT solution (Supplementary Fig. S6). The molarity was varied from 0.3 M to 0.1 M as shown in Fig. S6a–c. As the molarity decreases, the PZT NPs become small and they transform from polycrystalline to epitaxial. The 0.1 M PZT solution leads to epitaxial PZT NPs which are ideal for observing the internal strain distribution (Fig. 4). Figure 4b is a magnified image of Fig. 4a, and shows on the atomic scale how PZT NPs nucleate and grow on the surface of mwCNTs. HRTEM shows that the PZT NPs size is about 5 nm. Figure 4c shows the epitaxial relationship of perovskite PZT NP unit cells (indicated by yellow dots) relative to unit cells of mwCNTs (indicated by green dots). Illustrations of PZT and mwCNT unit cells, based on lattice profiles, are shown in Figs 4d,e and S7. The lattice parameter of the mwCNTs (C‒C) is about 2.46 Å, which is similar to armchair type-CNTs (2.44 Å). In PZT NPs, the distance of the nearest lattice on the mwCNTs (1^st^ d_PZT_) is 2.71 Å, and that of the 4^th^ and 5^th^ lattice are 3.24, 4.07 Å, respectively. The change of lattice distance indicates a strain gradient, which may contribute to the enhancement of the NG properties of PZT-CNT films by adding flexoelectricity to piezoelectrictity (Supplementary Fig. S7).

**Figure 5:**
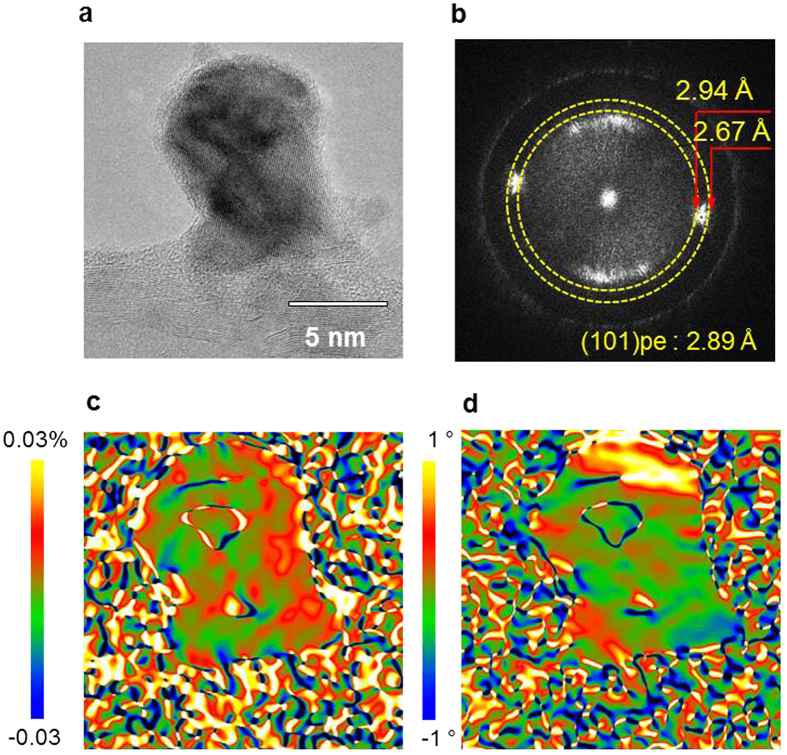
Geometrical phase analysis of PZT NP-CNTs. (**a**) HRTEM image, (**b**) Fourier-filtered power spectrum of PZT NP-CNTs grown at the end of mwCNTs, (**c**) lattice deformation and (**d**) rotation maps of PZT NP-CNTs.

Lattice distortion and rotation in the PZT NPs can be mapped at the atomic scale from HRTEM images using geometrical phase analysis (GPA). GPA generates quantitative lattice distortion and rotation maps from standard HRTEM images. Here GPA is used to quantify the local lattice shear strain of PZT NPs grown on mwCNTs, as shown in Fig. 5. Figure 5a shows HRTEM images of PZT NPs grown on the surface of mwCNTs and Fig. 5b displays the Fourier-filtered power spectrum of PZT NPs. In Fig. 5b, the lattice spacing of the (101) plane ranges from 2.67 to 2.94 Å. In the lattice distortion and rotation maps shown in Fig. 5c,d, the lattice strain of the PZT NPs on the near part of the mwCNTs is relaxed along the growth direction, which is consistent with the lattice distance change measured by HRTEM in Fig. 4.

## Discussion

We predicted that this enhancement of the NG properties is due to the following effects. First, direct growth of PZT crystals on mwCNTs improves the dispersibility of PZT-CNTs in PDMS. Well-dispersed PZT-CNTs avoid electrical shorting when ferroelectric domains are poled at high voltage and minimize leakage current when electrical output is generated by mechanical movement. This is consistent with the experimental observation of an NG based on a BTO NP-coated virus^8^. Second, electrical properties may be improved by the (001) preferred orientation of the PZT crystals on the mwCNTs, as shown in Fig. 2. Here, PZT-CNTs have a similar structure to PZT NP-CNTs, since they were subjected to the same stirring and heating process. This result is in agreement with previous work, which showed that the piezoelectric constant of PMN-PT with (001) texture is higher than that of randomly oriented PMN-PT^17^. Third, improvement in NG performance may result from the flexoelectric effect, i.e. electrical polarization caused by a strain gradient^18^. A recent study reported that the piezoelectric coefficient of flexible PZT nanomaterials increased due to the flexoelectric effect^10^. Therefore, we suggest that flexoelectric effects can also contribute to the enhancement of PZT-CNT NG properties.

In conclusion, we report the direct growth of PZT on mwCNTs and demonstrate piezoelectric NGs based on a PZT-CNT and PDMS matrix. PZT NP-CNTs are characterized by atomic-scale crystal PZT NPs on mwCNTs, as confirmed by FETEM analysis. Crystal orientation and density of PZT NPs on PZT NP-CNTs can be controlled by varying the molarity of PZT solution. Growth of PZT on mwCNTs caused an enhancement of NG output voltage compared to a simple mixture of PZT and mwCNTs. Prototype NGs based on PZT-CNTs repeatedly generated a voltage output of 8.6 V and a current output of 47 nA at a mechanical force of 20 N, approximately ten times higher than mixed PZT and mwCNTs. These results represent a significant step toward the application of NGs in flexible electronics, portable devices, and mechanical sensors.

## Methods

### Pretreatment of mwCNTs

As-grown mwCNTs (Cabon Nano-materials Technology, Korea) contain a variety of impurities including amorphous carbon, carbonaceous impurities, and metal catalyst particles. The pretreatment procedure of mwCNTs consisted of three steps. As-grown mwCNTs were oxidized at 550 °C in air to remove the carbonaceous impurities. Then, oxidized mwCNTs were immersed in a 6 M HCl solution with a ratio of 0.1 wt% and stirred at room temperature. This is referred to as the purification step. After purification, mwCNTs were neutralized with deionized (DI) water using centrifugation and dried on the hot plate at 80 °C. Purified mwCNTs shorten and become dispersive with open ends by chemical-etching of the amorphous carbon. HCl-treated mwCNTs were immersed in a HNO_3_/H_2_SO_4_ solution and stirred at 80 °C to functionalize them. Finally the mwCNTs were neutralized again with DI water and dried. The functionalization treatment creates structural defects and the formation of various organic groups such as carboxylic (-COOH), carbonyl (-C = O), and hydroxyl (-COH).

### Preparation of PZT-CNTs for NGs

PZT-CNTs were synthesized by refluxing, drying, and annealing a mixed solution including PZT NP-CNTs and PZT. The detailed process for the fabrication of PZT-CNTs is shown in Supplementary Fig. S1. PZT NP-CNTs were prepared by refluxing and filtering a mixed solution including PZT and functionalized mwCNTs with a syringe filter. A homogeneous 0.3 M PZT precursor solution was prepared via a modified 2-methoxyethanol-based sol-gel process^19^. The functionalized mwCNTs were sonicated into the precursor solution until a good suspension was obtained. The mixed solution was heated at 60 °C for 36 h and stirred via a magnetic bar in a two-necked round-bottom flask equipped with a water-cooled reflux condenser (a reflux process) to seed the PZT decorating the surface of mwCNTs. The filtered powder was dried overnight at 80 °C, ground to a fine powder using a mortar and pestle, pyrolyzed at 400 °C in air and annealed at 500‒700 °C in a nitrogen atmosphere. PZT NP-CNT powder was again mixed and refluxed under the same conditions as the previous process. Here, the weight ratio of CNT to PZT was 1:15. The solution was dried overnight at 80 °C in the evaporating dish, and heated to 500‒700 °C in a nitrogen atmosphere to induce crystallization. The dried PZT-CNT powder was then scraped off the dish using a flat spatula, ground using a mortar and pestle, pyrolyzed at 400 °C in air, and annealed at 500‒700 °C in a nitrogen atmosphere.

### Measurement of PZT-CNT NGs

Flexible PZT-CNT films were fabricated by mixing PZT-CNT powder into PDMS via the mechanical rambling method. Here, the weight ratio of the powder/PDMS was 15:85. The mixed powder/PDMS was spin-coated onto the flexible plastic substrate to form a film approximately 200‒300 μm thick. After drying, the coated films were peeled off and sandwiched between two Au/Cr-deposited polyimide films. In order to confirm the effect of PZT decorating the mwCNTs, mixed PZT and CNT films were also prepared. To enhance the piezoelectric effect of films, we applied a 2.5 kV mm^−1^ DC electric field across the electrodes at 150 °C for 48 h for electrical polarity. Output voltage and current were measured using a Keithley 2410 source meter, applying a periodic force with a 0.3 Hz repetition rate using a linear motor equipped with a Teflon stack.

### Characterization of PZT NPs-CNTs

Microstructural characteristics of PZT-CNTs and PZT NP-CNTs were investigated using FETEM and FESEM. Thermogravimetric analysis (TGA) was carried out using SDT-Q600 at a 10 °C min^−1^ heating rate under air flow (200 ml/min). The crystal structure was analyzed by SAED and Raman spectroscopy (Nanofinder 30; KBSI Jeonju Center, Korea).

## Additional Information

**How to cite this article**: Han, J. K. *et al.* Nanogenerators consisting of direct-grown piezoelectrics on multi-walled carbon nanotubes using flexoelectric effects. *Sci. Rep.* **6**, 29562; doi: 10.1038/srep29562 (2016).

## Supplementary Material

Supplementary Information
