# Supplementary material for: Nanogenerators consisting of direct-grown piezoelectrics on multi-walled carbon nanotubes using flexoelectric effects
Source: Sci Rep. 2016 Jul 13;6:29562. doi: 10.1038/srep29562 (PMC4942770; doi:10.1038/srep29562)
Supplement: Supplementary Information [file srep29562-s1.doc]

Supplementary Information:

Nanogenerators consisting of direct-grown-piezoelectrics on multi-walled carbon nanotubes using flexoelectric effects

Jin Kyu Han^1,2^ , Do Hyun Jeon^1^, Sam Yeon Cho^1,3^, Sin Wook Kang^1,3^, Sun A Yang, Sang Don Bu^1,3^, Sung Myung^2^, Jongsun Lim^2^, Moonkang Choi^4^, Minbaek Lee^4^, Min Ku Lee^5^

^1^Department of Physics, Chonbuk National University, Jeonju 54896, Korea

^2^Thin Film Materials Research Center, Korea Research Institute of Chemical Technology (KRICT), Daejeon 34114, Korea

^3^Research Institute of Physics and Chemistry, Chonbuk National University, Jeonju 54896, Korea

^4^Department of Physics, Inha University, Incheon 22212, Korea

^5^Nuclear Materials Development Division, Korea Atomic Energy Research Institute, Daejeon 34057, Korea

Correspondence and requests for materials should be addressed to S. D. Bu and J. Lim (email: sbu@jbnu.ac.kr, jslim@krict.re.kr ).

**Figure S1.** Schematic diagram of the synthesis procedure of PZT-CNTs (a) mix the acid-treated mwCNTs into the PZT precursor, (b) reflux the mixed solution, (c) filter the solution using a syringe filter, (d) dry and anneal the solution, (e) mix the PZT NP-CNT into the PZT precursor, (f) reflux the mixed solution, (g) dry and anneal the solution. Inset: FESEM images showing PZT NP-CNTs and PZT-CNTs.


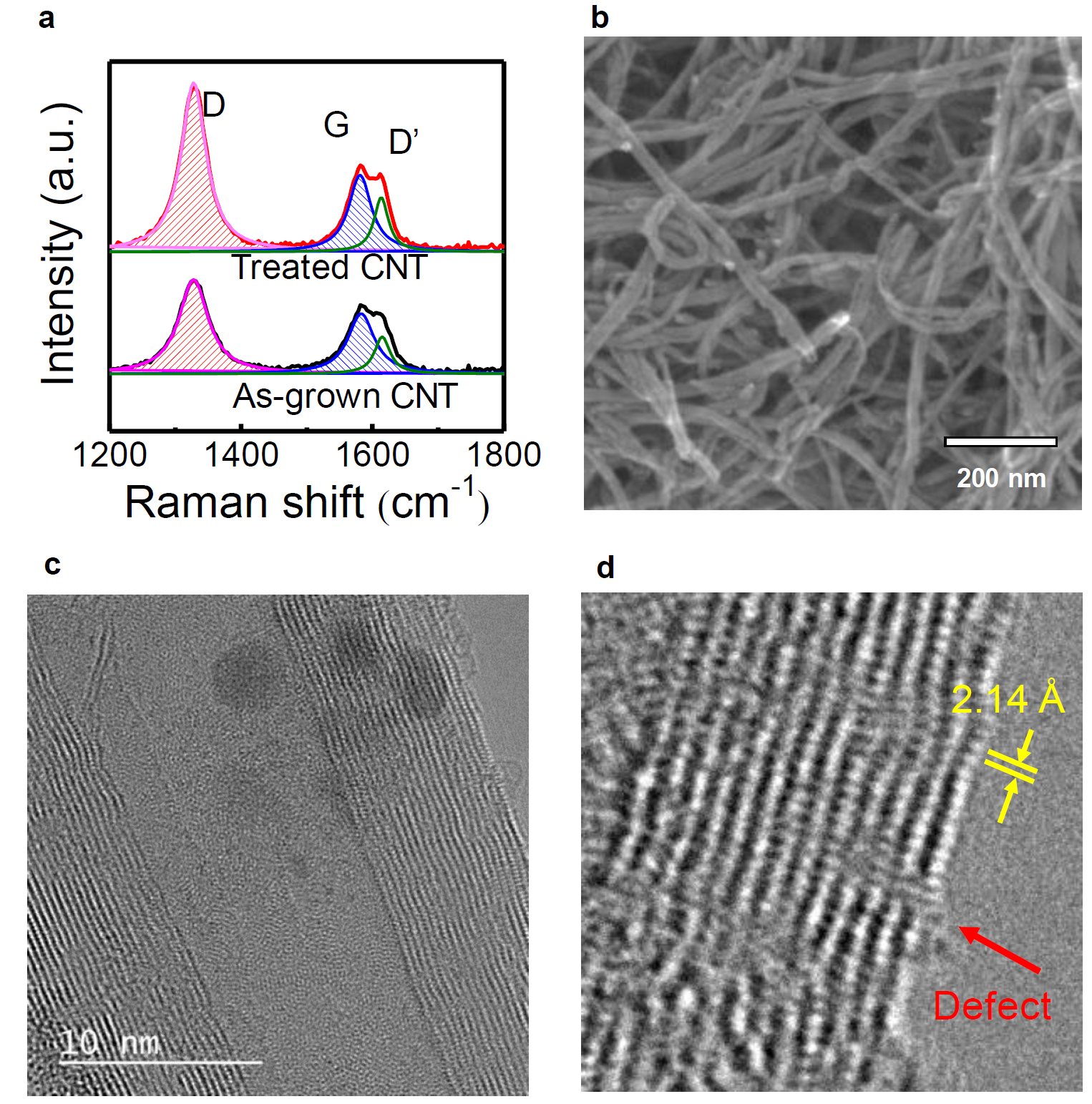


**Figure S2.** (a) Raman spectrum of the as-grown CNTs and acid-treated mwCNTs, (b) FESEM images, (c) FETEM images, (d) magnified FETEM image showing defect and lattice spacings.

**Table S1.** Lattice spacing, radius, and volume of crystallites PZT NP.

| Number | lattice spacing^*^ (Ǻ) | radius (nm) | volume (nm^3^) |
| --- | --- | --- | --- |
| Perovskite (JCPDS No. 33–0784) | |  |  |
| 1 | 2.89 [101] | 4.28 | 105.26 |
| 2 | 2.89 [101] | 3.53 | 59.10 |
| 3 | 2.89 [101] | 4.16 | 96.07 |
| 4 | 2.89 [101] | 4.07 | 90.25 |
| 5 | 2.89 [101] | 2.85 | 30.95 |
| 6 | 2.89 [101] | 2.96 | 34.58 |
| 7 | 2.85 [110] | 3.23 | 45.30 |
| 8 | 2.89 [101] | 5.53 | 225.97 |
| 9 | 2.85 [110] | 4.82 | 149.87 |
| Pyrochlore (JCPDS No. 26–0142) | | | |
| 10 | 2.39 [331] | 4.91 | 158.01 |

**Figure S3.** (a) FETEM image of PZT NP grown on mwCNT and (b) EDS line profile on the Pb, Zr, Ti, and O elements along the yellow line in (a). In order to clarify the presence of oxygen in PZT NPs, TEM-EDS analysis has been done. The atomic ratio of oxygen in the center of PZT NP obtained from the TEM-EDS line profile is about 54.7 %, which is a little bit smaller than that (60.0 %) of standard bulk sample. Based on the analysis, we can expect a small amount of the oxygen-deficient secondary phase in the PZT NPs, which is consistent with the TEM analysis results as shown in Fig. 2(f). These results strongly suggest that this work on PZT phase is very clear.

**Figure S4.** (a) XPS survey spectra and (b) O 1s spectra of the as grown CNTs, functionalized CNTs, and PZT NP-CNTs as a function of annealing temperature. The intensity of the peak associated with C=O bond decreases as the annealing temperature increases. In addition, between 200‒400 ºC, the peak disappears. Instead of that, the peak related with the oxygen absorbed into the surface of PZT NPs appears at 400 ºC. Besides, the peak associated with the lattice oxygen of PZT increases. Therefore, we can conclude that something having oxygen diffuse into the PZT NPs during the heat treatment so it is oxygenated, which is similar with the diffusion of carboxyl groups into oxygen vacancies in the sample of the ZnO-coated CNTs^16^.

**Figure S5.** Schematic diagram on synthesis of PZT-CNTs flexible film for nanogenerator measurement.


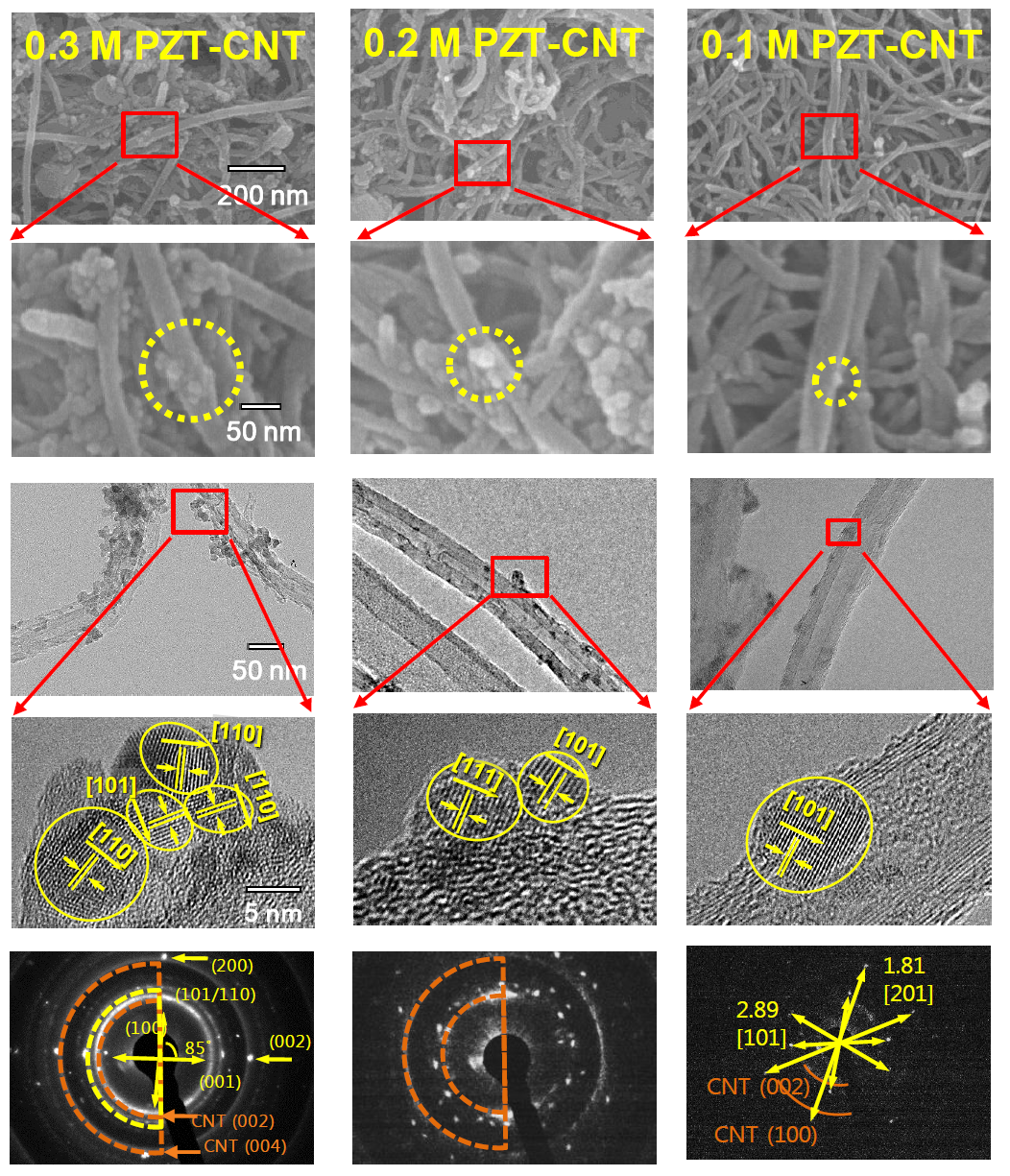


**Figure S6.** FESEM, FETEM, SAED patterns of the PZTNP-CNTs controlled the PZT molarity from 0.3 M to 0.1 M.

**Figure S7.** Schematic diagram of the lattice mismatch on the mwCNTs and PZT.
